# Optimal sequence for chain matrix multiplication using evolutionary algorithm

**DOI:** 10.7717/peerj-cs.395

**Published:** 2021-02-26

**Authors:** Umer Iqbal, Ijaz Ali Shoukat, Ihsan Elahi, Afshan Kanwal, Bakhtawar Farrukh, Mohammed A. Alqahtani, Abdul Rauf, Jehad Saad Alqurni

**Affiliations:** 1 Riphah College of Computing, Riphah International University Faisalabad Campus, Faisalabad, Pakistan; 2 Department of Mathematics, COMSATS Institute of Information Technology, Sahiwal Campus, Sahiwal, Pakistan; 3 Department of Computer Information Systems, College of Computer Science and Information Technology, Imam Abdulrahman Bin Faisal University, Dammam, Saudi Arabia; 4 Department of Educational Technology, College of Education, Imam Abdulrahman Bin Faisal University, Dammam, Saudi Arabia

**Keywords:** Chain matrix multiplication, Evolutionary algorithm

## Abstract

The Chain Matrix Multiplication Problem (CMMP) is an optimization problem that helps to find the optimal way of parenthesization for Chain Matrix Multiplication (CMM). This problem arises in various scientific applications such as in electronics, robotics, mathematical programing, and cryptography. For CMMP the researchers have proposed various techniques such as dynamic approach, arithmetic approach, and sequential multiplication. However, these techniques are deficient for providing optimal results for CMMP in terms of computational time and significant amount of scalar multiplication. In this article, we proposed a new model to minimize the Chain Matrix Multiplication (CMM) operations based on group counseling optimizer (GCO). Our experimental results and their analysis show that the proposed GCO model has achieved significant reduction of time with efficient speed when compared with sequential chain matrix multiplication approach. The proposed model provides good performance and reduces the multiplication operations varying from 45% to 96% when compared with sequential multiplication. Moreover, we evaluate our results with the best known dynamic programing and arithmetic multiplication approaches, which clearly demonstrate that proposed model outperforms in terms of computational time and space complexity.

## Introduction

Optimization means to find the optimal and diverse solution for a complex problem (Bengio, Lodi & Prouvost, 2020). There are many complex problems exist in the real life, it is difficult to solve these problems by divination. In these problems, the resources are limited, which lead to many constraints. Optimization plays an important role to solve these problems, because optimization uses the resources in efficient way. These complex problems have many scenarios where an objective can be transformed into an optimization problem. Optimization problems are classified into two types: single-objective optimization problem and multi-objective optimization problem. For optimization problems the researchers proposed many evolutionary algorithms like Genetic Algorithm (GA) (Deb et al., 2002; Waheeb & Ghazali, 2019), Dynamic Evolution (DE) (Storn & Price, 1997), Evolutionary Strategies (ES) (Huning, 1976), Ant Colony Optimization (ACO) (Dorigo, Maniezzo & Colorni, 1996), Genetic Programming (GP) (Mugambi & Hunter, 2003), Evolutionary Programming (EP) (Coello, Pulido & Lechuga, 2004), Particle Swarm Optimization (PSO) (Huang, Suganthan & Liang, 2006), Group Counseling Optimizer (GCO) (Eita & Fahmy, 2010), Multi-Objective Group Counseling Optimizer (MOGCO) (Ali & Khan, 2013) and Constraint Group Counseling Optimizer (CGCO) (Eita, Shoukry & Iba, 2014). These evolutionary algorithms have been effectively used to solve science and engineering optimization problems such as feature selection (Zhou, Liu & Chen, 2011), intrusion detection (Gómez et al., 2013), optimal security hardening (Dewri et al., 2012), and dynamic risk management (Poolsappasit, Dewri & Ray, 2011), etc. However, no one paid attention to applying these algorithms in the field of chain matric multiplication with the exception of the Genetic Algorithm (GA). But GA belongs to the population based branch of evolutionary algorithms.

There are the two types of evolutionary algorithms: first is the population based evolutionary algorithms and, second is the evolution based evolutionary algorithms. The evolution based algorithms are faster that the population based algorithms (Ali & Khan, 2013), because the population based algorithms maintain the record of all individuals from start to end of the process, but the evolution based algorithms update the individuals table after each iteration and maintain the record of best one individual from both child and parent. The GCO, CGCO and MMOGCO are the evolution based algorithms. This is why these algorithms are fast in terms of computational time as compare to other evolutionary algorithm (Ali & Khan, 2013). In this work, we have selected the GCO algorithm for the proposed model because this algorithm uses for both dominated and non-dominated data set, and used for single objective optimization problems. The CGCO used for only dominate data sets, this is not useful for non-dominate data sets. The MOGCO uses for the multi-objective optimization problems.

**Figure 1:**
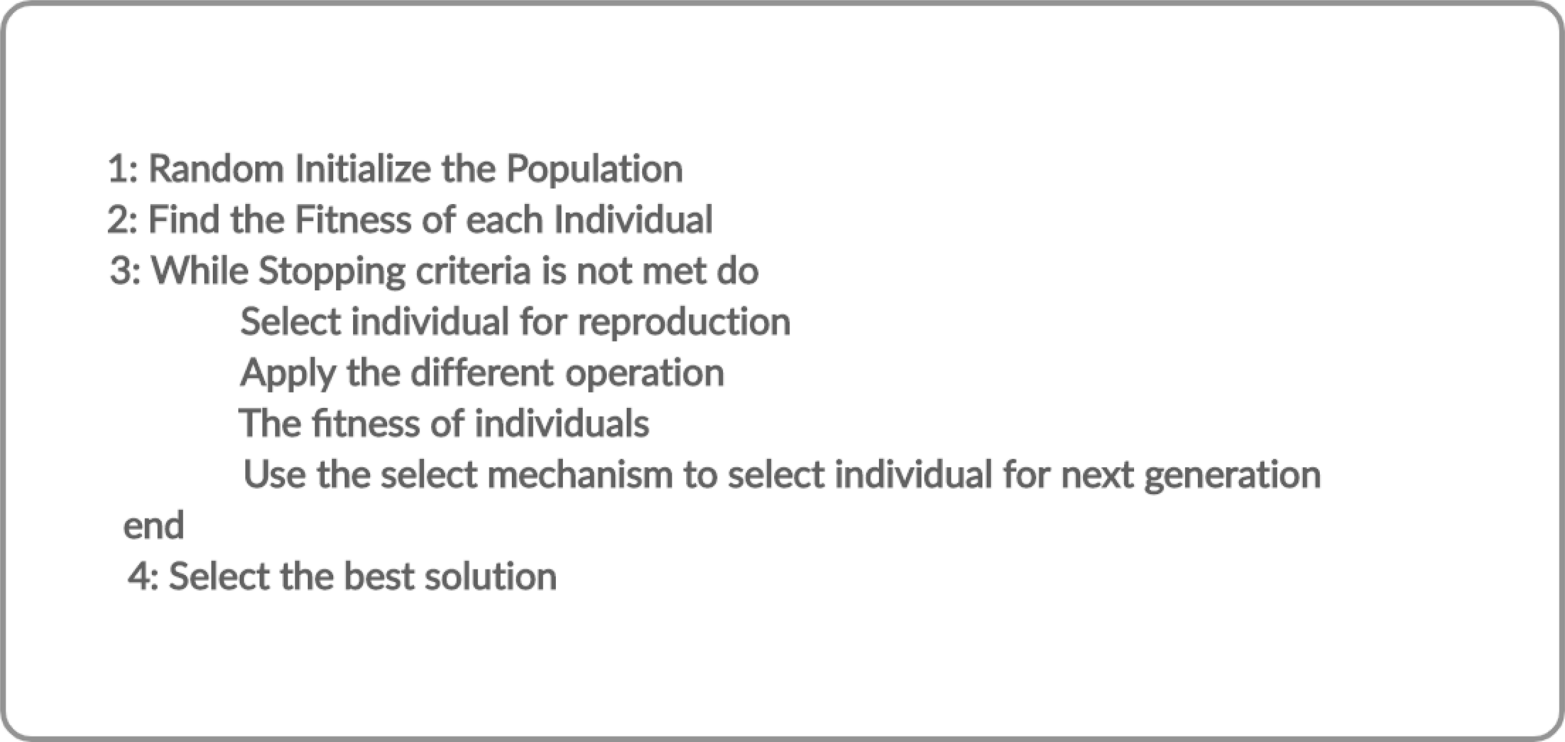
Outline of evolutionary algorithms.

Evolutionary algorithms build solutions that are more fit according to the desired properties of design problems. Commonly these algorithms used to generate the high level solutions of optimization and search problems likely mutation, crossover and selection. Further, these algorithms used a method of randomly selection solution known as the initiatory population and form the new population using different operations. The general outline of evolutionary algorithms (Dewri et al., 2012) is shown in the Fig. 1.

Matrix Multiplications (MM) and Chain Matrix Multiplication (CMM) are two different types of operations. Matrix Multiplication is a binary operation in mathematics, in which we produce a matrix from two/more matrices (O’Connor & Robertson, 2019). “The rule of matrix multiplication, in which the number of columns in the first matrix must be equal to the number of rows in the second matrix” (Srivastava et al., 2020). The result of these two matrices known as matrix product. MM was initially designed to represent the composition of linear maps. Therefore, MM is a basic tool of linear algebra, and as such has several applications in different areas of applied mathematics and also in mathematics, physics, and engineering. The computation of matrix products is a fundamental operation in all computation applications of linear algebra. MM is a binary operation in which we produce the result from two matrices in a new matrix (Mishra et al., 2020), whereas, CMM is a sequence of matrices in which we find the most efficient way to multiply a sequence of matrices, to decide which order to accomplish the multiplications. We only defined the number of operations to multiply the matrices.

**Figure 2:**
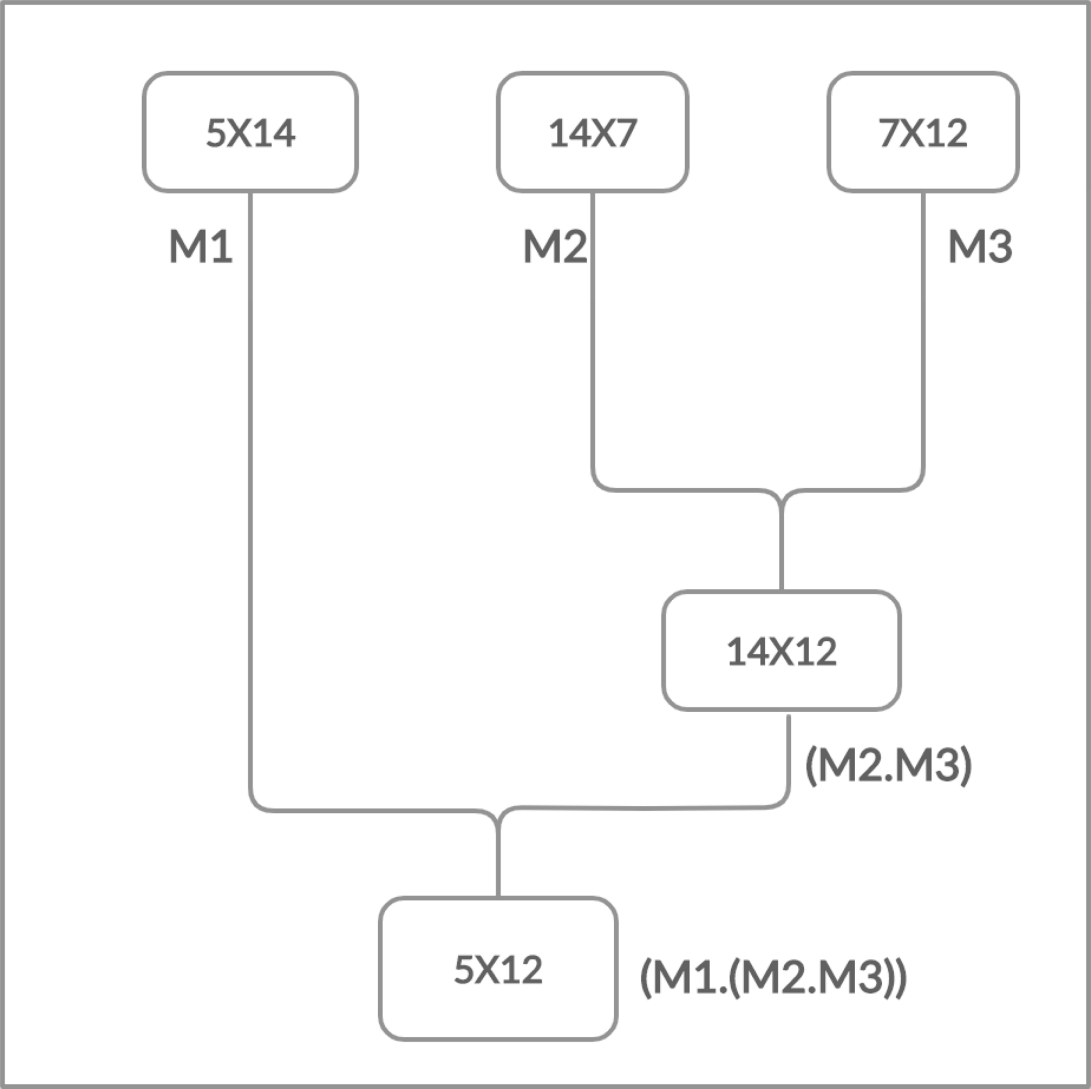
Multiplying right two matrices first.

**Figure 3:**
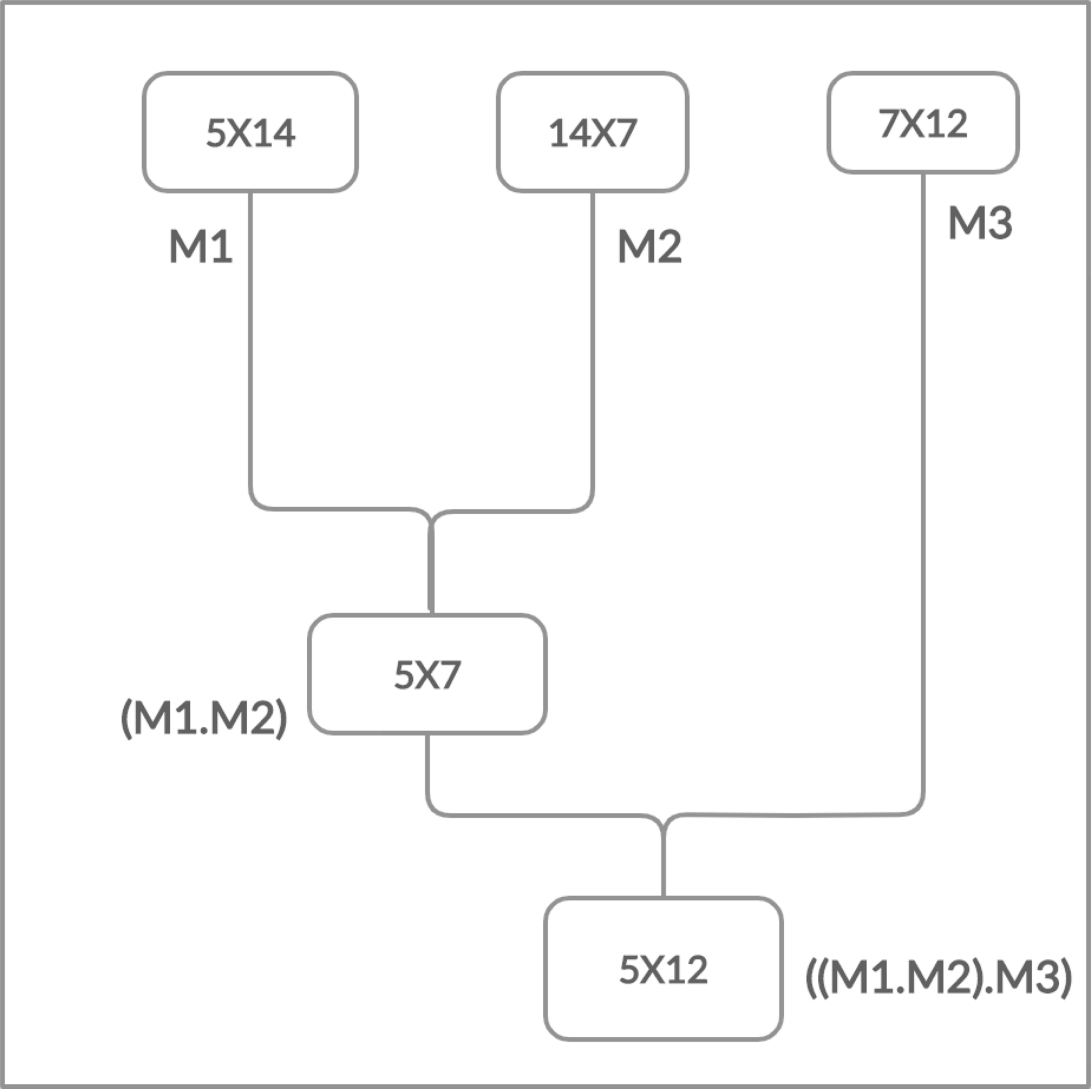
Multiplying left two matrices first.

Moreover, the matrices have the cost which is determined in the form of rows and columns (p × q). The matrix multiplication is totally depends on this cost. The multiplication is possible if and only if the number of columns of first matrix is equal to the number of rows of second matrix. Chain Matrix multiplication is an associative operation, the chain matrix multiplication order does not affect the final result but it can affect the total number of performed operations as shown in Figs. 2 and 3.

In this article, we have proposed an efficient Group Counseling Optimization (GCO) algorithm based model. The main contributions made by this article are as follows:

- The proposed model implemented the GCO algorithm to for CMM problems. It finds out the optimal sequence for CMM.
- The comparison of proposed model has done with the existing techniques such as dynamic programing approach, arithmetic multiplication approach and sequential approach based on space complexity, time complexity and number of multiplication operations.

The rest of the article is organized as follows. “Related Work” summarizes the related work and reviews the literature on evolutionary algorithms and techniques used for the CMMP. In “Proposed Model”, we explain the proposed model in detail. “Experimental Design” discusses the experimental design. “Tool and Technology”, discuss the tool and technologies. “Results and Discussions” presents experimental results and comparison of proposed model with existing techniques for CMMP. “Concluding Remarks” concludes this research work.

## Related Work

**Figure 4:**
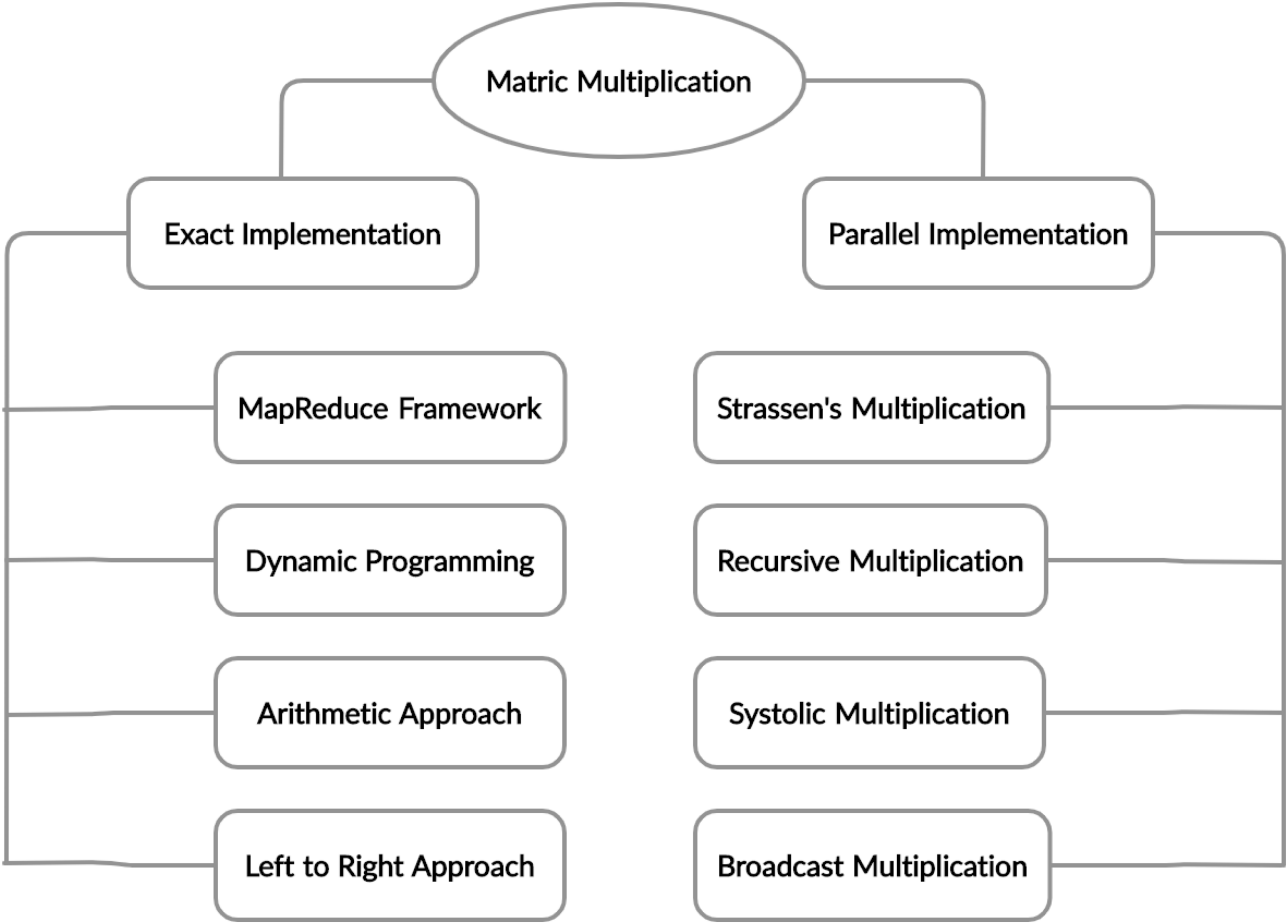
Taxonomy of matrix multiplication.

Chain Matrix multiplication is an associative operation, the chain matrix multiplication order does not affect the final result but it can affect the total number of performed operations. Different architectures and techniques were proposed to solve this problem, which is shown in Fig. 4 and the summary of literature review discussed in Table 1.

**Table 1:** Summary of literature review.

SR#	Title	Techniques	Limitations	References
1	Genetic Algorithm	Genetic Algorithm, Random Selection, Performance Matrices	The Genetic Algorithm is the population base algorithm	Mirjalili (2019)
2	Constrained Group Counseling Optimization	Performance Matrices, Single-Objective Functions, Single-Objective Optimization Problems, Group Counseling Optimizer, Random Selection	Used the Dominate Data Set, Social Problems of Human	Eita & Fahmy (2010)
3	Experiments with a software component enabling NetSolve with direct communications in a non-intrusive and incremental way	Expression based approach, Binary Tree Method	The main issue of this approach is that the execution time increases for large grid matrix multiplications	Zuo & Lastovetsky (2007)
4	Accelerating the dynamic programing for the matrix chain product on the GPU	Dynamic Programming Approach	It is time consuming because time varies with *n*^3^ here *n* is number of matrices	Nishida, Ito & Nakano (2011)
5	Hama: An efficient matrix computation with the map reduce framework	Map Reduce, Binary Tree	The main issue of map reduce technique is that size of matrix does not fit in the memory and difficult to optimize the multi-way join operation in Map Key if the same number assigned to more attributes	Seo et al. (2010)
6	Matrix-Chain Multiplication Using Greedy and Divide-Conquer approach	Greedy Approach, Genetic Algorithm	The greedy approach stuck at the local optimal and consider it global optimal	Lakhotia et al. (2015)
7	A Chain-Multiplier for Large Scale Matrix Multiplication	Systolic Approach, Dynamic Programming	The main issue of this approach is that there are limited hardware resources	Wei et al. (2017)
8	Matrix chain multiplication via multi-way join algorithms in Map Reduce	Map Reduce framework	Size of matrix does not fit in the memory	Myung & Lee (2012)
9	Theoretical and Experimental Study of a Parallel Algorithm Solving the Matrix Chain Product Problem	Three phase methodology based on dynamic programing	Short matrix length the execution time is much larger	Mabrouk, Hasni & Mahjoub (2017)

There as multiple approaches used for CMMP like: dynamic programing approach (Ben Charrada, Ezouaoui & Mahjoub, 2011), sequential approach (Kung, 1982, 1980), greedy approach (Lakhotia et al., 2015) and arithmetic approach (Hafeez et al., 2007). According to the literature, the dynamic programing approach and arithmetic approach for the CMMP provides the optimal results but the problem is that these approaches are time consuming and required the more space. According to the literature it is also stated that the greedy approach provides optimal sequence for the CMM in some case but mostly provided the sequence for CMM which one perform the more multiplication operations, because the greedy approach stuck at local optima. That’s why greedy approach only used for the small data set where the local optima is the global optima. The sequential multiplication is well known approach used for the CMMP, but the sequential approach failed to provides the optimal sequence for CMM and it is also time consuming approach and required more space.

The product of chain matrix multiplication can be acquired by using the standard tree method that was proposed by Zuo & Lastovetsky (2007). The product of A1, A2, A3…, A8 can be obtained by using binary tree method. Input matrices A1, A2, A3…, A8 are input from the leaves and tree will compute the final result A12345678 at the root. Direct communications are enabled between these four servers by directly transferring output A12 from node 1 to node 2, output A56 from node 3 to node 4, output A1234 from node 2 to node 4. The binary tree built such that the root are at the bottom level and leaves at the top level. Each particular node corresponds to a matrix product and the leaves corresponds the product of two successive matrices of the chain matrix. The root corresponds the final results of the given sequence of matrices. The main issue of this approach is that the execution time increases for large grid matrix multiplications.

MapReduce is a programing technique and a programing model which was designed for distributed computing (Seo et al., 2010). This technique consists of two important tasks that is Map and Reduce. Map function takes the set of data and converts it into individuals elements and broken down into tuples. Reduce task proceeds the output from the map function as input then associations those tuples into a small set of tuples. For the large matrix multiplication, Myung and Jaeseok proposed an implementation based MapReduce framework (Myung & Lee, 2012). They expanded a binary multiplication problem to n-ary multiplication for joining the several matrices operation and represented a matrix which is consists of records (row, col, and val). The main issue of mapreduce technique is that size of matrix does not fit in the memory and difficult to optimize the multi-way join operation in MapKey if the same number assigned to more attributes.

A Dynamic Programming Algorithm approach was proposed to solve the large complex problems in Nishida, Ito & Nakano (2011) which is work like divide and conquer principle. In dynamic programing a recursive function is defined to get the optimized parentheses which give the minimum number of multiplications. In this approach, the original chain splitting into sub-chain of length such that the product (*A_i_*…*A_k_*) (*A_k_*_ + 1_…*A_j_*). A function (Tithi et al., 2015) *w* (*i*, *k*, *j*) is used which proceeds the cost of parenthesis combination of (*A_i_*…*A_k_*) and (*A_k_*…*A^j^*). This algorithm allocates a cost to all products and then stores the best solution together with its cost. It will compute the matrices will all possible ways of multiplication with each other and store them in a table, and gives the optimal sequence at the end. The main issue of this approach is that the problem size is held fixed

Graphics Processing Units (GPUs) built approach was proposed and tested using C++ AMP on NVIDIA GPUs (Shyamala, Kiran & Rajeshwari, 2017). In this approach two types of functions are used in C++ AMP, A Pre-Processing function which is used for the multiplication number calculation with minimum number of multiplication operations of matrices is chooses for GPU computing. A Matrix Multiplication Parallel function is used to observes for keyword (restrict (amp)) to be executed to get the code on GPU. The drawback of this approach is that it has limited sized matrices numbers concurrently runs with different values (e.g., 3 × 4). The proposed work is implemented with an integrated graphics card.

Using the greedy approach a solution was proposed to determine the minimum number of multiplication operations (Lakhotia et al., 2015). In this study, they modify the greedy approach with divide and conquer approach and the main idea behind this modification is to solve the multiplication problems in a top down fashion. They take an input in array order *p*[0….*n*], and divide the *p* array into *n* sub-array. Each sub-array consists at least one or at most 2 elements. This process was done in a greedy way, at each step only one least element is selected among all elements in the array *p*. So that, the cost of multiplication kept minimum at each single step. This approach ensures that the result is optimal with minimum cost consists and the output was a fully parenthesized of matrices. This algorithm did not chosen the correct least value when the dimensions of matrices are same.

Many algorithms and methods are proposed for better performance using Strassen’s implementation. Strassen’s algorithm known as Dynamic General Fast Matrix Multiplication (DGEFMM) algorithm which was used for any size of matrix with minimum number of scalar multiplication using minimum storage (Benson & Ballard, 2015). Matrix multiplication operations are more expensive than the matrix addition, this tradeoff is known as faster algorithms. Fast Strassen’s algorithm follows the same block structure as recursive multiplication with seven matrix multiplications and 18 additions.

A hardware accelerator systolic suitable architecture “(point to point multiplication operation is used between all interrelated processing elements)” for large scale matrix multiplication was proposed (Zuo et al., 2017), it is very suitable for hardware design and requires lower bandwidth than systolic structure. The drawback of this approach is problematic to complete the whole matrix operations at a time due to limited hardware resources. It is essential to divide the matrix into small portions, and multiply each of the small chunks with the others chunks. The chain multiplier is able to handle the block matrix multiplication well. The main issue of this approach is that there are limited hardware resources.

For CMMP Mabrouk, Hasni & Mahjoub (2017) proposed Dynamic Programming based three phase approach. The Dynamic Programing provides the optimal sequence (parenthesization) for chain matrix multiplication problems, but it is time consuming because time varies with *n*^3^ here *n* is number of matrices.

Henrik Barthel and Marcin were designed a new approach (Barthels, Copik & Bientinesi, 2018) based on expressions. These expressions consists of the products of vectors and matrices. These expressions are mapped onto a computational kernel set of K. Additionally; the mapping of expression has to minimize a user-selected expense metric “(such as number of flops or execution time).” The output is then a sequence of kernel calls that computes the original expression. The main issue of this approach is that the type of pattern matching CLAK kernel uses is expensive.

## Proposed Model

**Figure 5:**
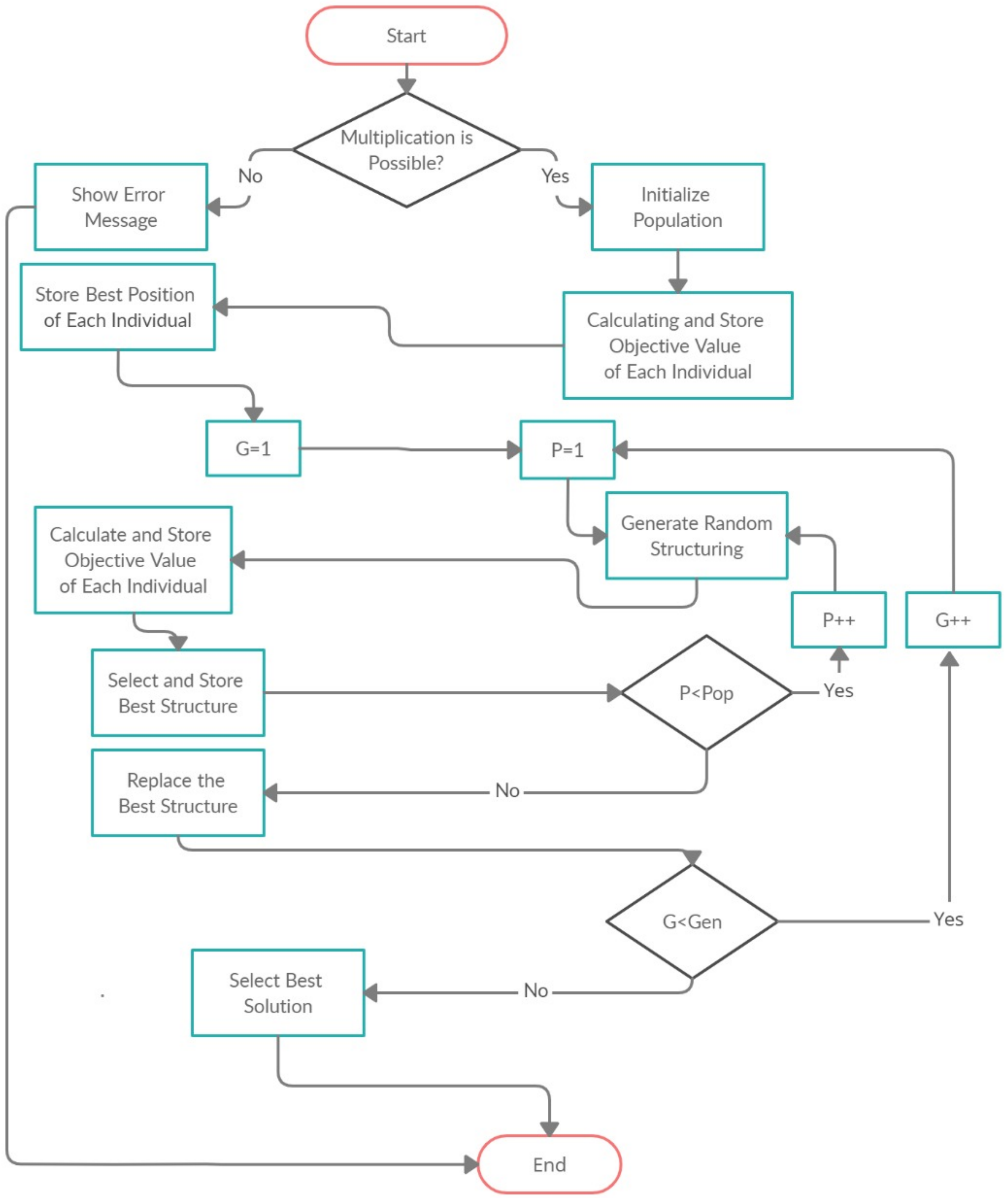
Flow chart of proposed model.

**Figure 6:**
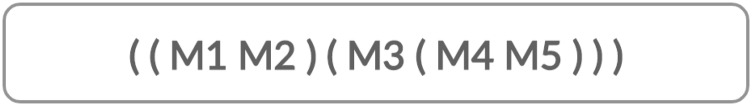
Chromosomes.

The proposed model based on the Group Counseling Optimizer (GCO) (Eita & Fahmy, 2014) algorithm in which we generate the parenthesis for the CMM to minimize the CMM operations (scalar products). The flow chart of proposed model is shown in Fig. 5. In Fig. 6 the Pop is the population, Gen is the generations, P and G also donated to population and generations respectively. The product of population and generations is the fitness evolution value like: If population is 100 and generations are 50 then the fitness evolution value is 5,000.

The model firstly takes the input file which contains the number of matrices, rows and columns. The model reads the data from the file and stores it in the string form. After that, model assigns the name to each matrix like *M*_1_, *M*_2_, and *M*_3_ and so on. After assigning the name to each matrix, proposed model check that the criteria for matrix multiplication. If multiplication is not possible, then model shows the error message, otherwise the model assign the random structuring sequence to the matrices as shown in the Table 2.

**Table 2:** Initialization of population.

No. of matrices	Sequence of dimensions	Parenthesis
5	9,7,10,90,12,40	(*M*_1_(*M*_2_((*M*_3_ *M*_4_)*M*_5_)))
5	9,7,10,90,12,40	((*M*_1_*M*_2_)(*M*_3_(*M*_4_ *M*_5_)))
5	9,7,10,90,12,40	(*M*_1_((*M*_2_ *M*_3_)(*M*_4_ *M*_5_)))
5	9,7,10,90,12,40	(((*M*_1_ *M*_2_)*M*_3_)(*M*_4_ *M*_5_))

Table 2 shows that, the population has the 4 individuals Each individual in the population is called chromosome and chromosome is the combination of gens as shown in Fig. 6.

After initialization of population, the model calculates the fitness value of each chromosome and stores it as shown in Table 3.

**Table 3:** Chromosomes fitness value.

No. of matrix	Sequence of dimensions	Random structuring sequence	Scalar product
5	9,7,10,90,12,40	(*M*_1_(*M*_2_((*M*_3_ *M*_4_)*M*_5_)))	20,920
5	9,7,10,90,12,40	((*M*_1_*M*_2_)(*M*_3_(*M*_4_ *M*_5_)))	83,430
5	9,7,10,90,12,40	(*M*_1_((*M*_2_ *M*_3_)(*M*_4_ *M*_5_)))	77,220
5	9,7,10,90,12,40	(((*M*_1_ *M*_2_)*M*_3_)(*M*_4_ *M*_5_))	84,330

After calculating and storing the fitness value of chromosomes, the model stores the chromosomes at their best position as shown in the Table 4.

**Table 4:** Chromosomes best position.

No. of matrix	Sequence of dimensions	Random structuring sequence	Scalar product
5	9,7,10,90,12,40	(*M*_1_(*M*_2_((*M*_3_ *M*_4_)*M*_5_)))	20,920
5	9,7,10,90,12,40	(*M*_1_((*M*_2_ *M*_3_)(*M*_4_ *M*_5_)))	77,220
5	9,7,10,90,12,40	((*M*_1_*M*_2_)(*M*_3_(*M*_4_ *M*_5_)))	83,430
5	9,7,10,90,12,40	(((*M*_1_ *M*_2_)*M*_3_)(*M*_4_ *M*_5_))	84,330

After storing the chromosomes at their best position, the model starts the process of reproduction of new chromosomes. In this process, the model firstly generate the multiple random structures for the matrices, then select the best structure from the generated structures on the bases of fitness value (scalar products) and store it in the column of corresponding parent chromosome as shown in the Table 5.

**Table 5:** Reproduction of chromosomes.

No. of matrix	Sequence of dimensions	Parent (Parenthesis)	Scalar multiplications	Child (Parenthesis)	Scalar multiplications
5	9,7,10,90,12,40	(*M*_1_(*M*_2_((*M*_3_*M*_4_)*M*_5_)))	20,920	((((*M*_1_*M*_2_)*M*_3_)*M*_4_)*M*_5_)	22,770
5	9,7,10,90,12,40	(*M*_1_((*M*_2_*M*_3_)(*M*_4_*M*_5_)))	77,220	(*M*_1_(*M*_2_(*M*_3_(*M*_4_*M*_5_))))	84,520
5	9,7,10,90,12,40	((*M*_1_*M*_2_)(*M*_3_(*M*_4_*M*_5_)))	83,430	(*M*_1_(((*M*_2_*M*_3_)*M*_4_)*M*_5_))	19,740
5	9,7,10,90,12,40	(((*M*_1_*M*_2_)*M*_3_)(*M*_4_*M*_5_))	84,330	((*M*_1_(*M*_2_(*M*_3_*M*_4_)))*M*_5_)	16,716

After reproduction of chromosomes, the model checks that which one is best from parent and child chromosomes, then select the best one chromosome and store in the generation table in the ascending order as shown in the Table 6.

**Table 6:** First generation.

No. of matrix	Sequence of dimensions	Parenthesis	Scalar products
5	9,7,10,90,12,40	((*M*_1_(*M*_2_(*M*_3_*M*_4_)))*M*_5_)	16,716
5	9,7,10,90,12,40	(*M*_1_(((*M*_2_*M*_3_)*M*_4_)*M*_5_))	19,740
5	9,7,10,90,12,40	(*M*_1_(*M*_2_((*M*_3_*M*_4_)*M*_5_)))	20,920
5	9,7,10,90,12,40	(*M*_1_((*M*_2_*M*_3_)(*M*_4_*M*_5_)))	77,220

After achieving the first generation the model use it for generating further generations. The generations are generated until the break point. After achieve the last generation, the model select the best solution from the last generation. The best solution is selected on the bases of scalar products, the chromosome which one has the minimum value of scalar products select as an optimal solution.

The fitness function decides that how fit a solution from the all generated solutions. The fitness function gives the score to each individuals. The selection probability of an individual is based on its fitness cost. High fitter chromosomes has high chances of survival to next generation, whereas, the worst fit chromosomes has low chances of survival. The fitness of the individuals is computed according to the following function:

(1) $$\mathop \sum \limits_{i = 1}^n X_i= X_1 + X_2 + X_3\ldots X_n$$

(2) $${\rm Where},\qquad X_i = (M_1. M_2)$$

(3) $${\rm And}\qquad X_{i+1} = M_1. (M_2. M_3)\ {\rm or}\ (M_1. M_2). M_3$$

The fitness function applied to compute the cost of all individuals and compared with the whole population. Furthermore, then sort the population according to its fitness score. The minimum score known as the best individual in the population and has high probability to survive the next generation and sorting them from best to worst order. With the use of stack implementation compute the cost (fitness) of each matrix string.

For example:

We have three number of matrix: ((*M*_1_ *M*_2_) *M*_3_)

*M*_1_ = 5 × 10

*M*_2_ = 10 × 15

*M*_3_ = 15 × 20

So, the fitness of above individuals:

(4) $$X_1 = M_1 M_2 = 5 \times 10 \times 15 = 750$$

(5) $$X_2 = X_1. M_3 = 5 \times 15 \times 20 = 1,500$$

Total Fitness:

(6) $$X_1 + X_2 = 750 + 1,500 = 2,250$$

Performance of this work in the form of cost which increase the overall efficiency of Chain Matrix Multiplications. In optimization, the cost is the continuous process of getting the best results with no impact on the system and guaranteeing the system satisfaction scores are sustained. In chain matrix multiplication, the goal is to find the most efficient way to multiply the matrices. The multiplication order that minimizes the total number of required operations to reduce the overall cost of CMM.

## Experimental Design

The evaluation of the proposed version of CMM compared with the existing approaches for CMM like dynamic programing approach for CMM, arithmetic approach for CMM, sequential multiplication approach for CMM. Results of the proposed model of CMM compared with the existing CMM approaches and represented the results. The behavior of some existing approaches has shown to observe how much performance is incremented and how it underutilizes the desires bandwidth. The behavior of existing approaches has been discussed in “Related Work”.

The data set is collected from different articles published by Ben Charrada, Ezouaoui & Mahjoub (2011), Hafeez et al. (2007) and Kung (1982, 1980). The senility analysis performed on this data. There are the following parameters of data set.

- Name of Matrices
- No. of Matrices
- No. of Rows of Matrices
- No. of Columns of Matrices

Rules of Matrix Multiplications, rules of Chain Matric Multiplication and sequence of Chain Matrix Multiplications (Shyamala, Kiran & Rajeshwari, 2017; Mabrouk, Hasni & Mahjoub, 2017; Barthels, Copik & Bientinesi, 2018; Srivastava et al., 2020), computational time (Coello, Pulido & Lechuga, 2004) and space complexity (Coello, Pulido & Lechuga, 2004) are also used in this research work.

## Tool and Technology

The experiments for the proposed computational model were implemented using MATLAB R2013b running on Microsoft Windows 10 64-bit OS. The PC was built with 8 GM Random Access Memory (RAM) and an Intel Core i5 2.30 GHz Central Processing Unit (CPU).

## Results and Discussions

The results of proposed model for optimal solution of CMM problems (CMMP) are demonstrated. The proposed model compared with the dynamic programing approach, sequential multiplication approach and arithmetic multiplication approach for the CMMP. So far we have demonstrated a GCO based model that computes the optimal cost for chain matrix multiplications. Table 7 summarizes the main results of time complexity and space complexity of different algorithms. In the Table 7 “*n*” is the number of matrices. The results show that proposed model outperform as compare to other techniques in terms of time complexity and space complexity.

**Table 7:** Time and space complexity of different approaches.

	Dynamic approach	Sequential multiplication	Arithmetic approach	GCO
Time complexity	(*n*^3^)	(*n*^2^)	(*n*^3^)	(*n*^2^)
Space complexity	(*n*^2^)	(*n*^2^)	(*n*^2^)	(*n*)

In the comparative tables, the dimension column has the values as: 10, 20, 30, 40, 50, 60. It’s mean that the first matrix has the dimensions (rows & columns) 10 & 20, the second matrix has the dimensions 20 & 30, the third matrix has dimensions 30 & 40 and so on.

The proposed model results are compared with sequential multiplication with matrix size varying from 0 to 50 as shown in Table 8. It is obvious that, there is significant amount of sequential multiplication reduction is proportional to the number of matrices and the sequence of dimensions that apply on the chain matrix multiplication. When we apply our proposed GCO model on the same data set, it is evidently demonstrated that there is 45–96% improvement comparatively sequential multiplication approach for up to 50 number of matrices respectively, where dimension size varying from 1 to 100. Table 8 also shows the relative improvement results with the optimal structure of parenthesization obtained by GCO proposed model. We get the results of sequential multiplication from the published articles (Kung, 1982, 1980) and compared the results with proposed model after apply the proposed model on the same data set. Moreover, the proposed model is better than the sequential multiplication in the terms of computational time and space as shown in Table 7.

**Table 8:** Comparison of proposed model with sequential multiplication.

No. of matrix	Sequence of dimensions	Optimal parenthesis	Sequential multiplication	GCO multiplication	Improvement
9	94,67,56,17,80,68,10,78,7,5	(M1*(M2*(M3*(M4*(M5*(M6*((M7*M8)*M9)))))))	1,273,230	98,220	92%
12	42,54,49,22,62,46,93,97,82,59,24,86,56	(((M1*(M2*M3))*((((((M4*M5)*M6)*M7)*M8)*M9)*M10))*(M11*M12))	1,777,734	970,214	45%
15	27,98,89,40,36,82,6,11,3,23,15,91,87,35,3,43	((M1*(M2*(M3*(M4*(M5*(M6*((M7*M8)*((M9*M10)*(M11*(M12*(M13*M14)))))))))))*M15)	816,480	101,322	88%
18	94,30,63,79,52,10,6,13,93,97,3,8,67,40,38,6,89,61,71	((M1*(M2*(M3*(M4*(M5*(M6*(M7*(M8*(M9*M10)))))))))*(((((((M11*M12)*M13)*M14)*M15)*M16)*M17)*M18))	3,518,984	139,845	96%
21	57,92,76,77,28,13,47,27,3,67,89,4,93,16,24,4,14,83,89,92,33,19	((M1*(M2*(M3*(M4*(M5*(M6*(M7*M8)))))))*((((((((((((M9*M10)*M11)*M12)*M13)*M14)*M15)*M16)*M17)*M18)*M19)*M20)*M21))	2,658,537	158,058	94%
24	79,68,62,22,98,35,62,99,21,39,91,79,81,31,11,4,87,90,90,72,57,92,3 6,72,59	((M1*(M2*(M3*(M4*(M5*(M6*(M7*(M8*(M9*(M10*(M11*(M12*(M13*(M14*M15))))))))))))))*((((((((M16*M17)*M18)*M19)*M20)*M21)*M22)*M23)*M24))	6,688,377	377,216	95%
30	50,44,56,33,44,5,9,10,12,22,32,26,41,28,19,29,41,23,18,25,22,34,33,13,33,11,43,21,24,56,71	((M1*(M2*(M3*(M4*M5))))*((((((((((((((((((((((((M6*M7)*M8)*M9)*M10)*M11)*M12)*M13)*M14)*M15)*M16)*M17)*M18)*M19)*M20)*M21)*M22)*M23)*M24)*M25)*M26)*M27)*M28)*M29)*M30))	1,258,650	153,290	88%
50	56,34,33,46,39,50,65,32,10,15,30,24,25,13,7,11,19,30,15,3,20,31,50,9,10,16,44,22,10,16,44,22,10,19,30,40,45,23,22,14,30,11,22,24,32,15,19,29,34,5,9,23,29,34,9	((M1*(M2*(M3*(M4*(M5*(M6*(M7*(M8*(M9*(M10*(M11*(M12*(M13*(M14*(M15*(M16*(M17*(M18*M19))))))))))))))))))*((((((((((((((((((((((((((((((((((M20*M21)*M22)*M23)*M24)*M25)*M26)*M27)*M28)*M29)*M30)*M31)*M32)*M33)*M34)*M35)*M36)*M37)*M38)*M39)*M40)*M41)*M42)*M43)*M44)*M45)*M46)*M47)*M48)*M49)*M50)*M51)*M52)*M53)*M54))	1,969,800	112,434	93%

To check and investigate the performance of our new proposed model, we compare it with dynamic programing. The comparison between proposed model and Dynamic Programming demonstrated in Table 9, in this table the results show that the both proposed model and dynamic approach provides the same results. Because the dynamic approach always provides the optimal result of problems, so we can say that the proposed model provided the optimal results. We get the results of dynamic approach from the published article (Ben Charrada, Ezouaoui & Mahjoub, 2011), which also explain that the dynamic approach provides the optimal result for the used data set, we get the same data set and apply the proposed model on the data that’s why Table 9 proves that the proposed model provides the optimal result. Table 7 describes that the results of proposed model outperforms as compare to dynamic programing in terms of computational time and space. So we can say that the proposed model provides the optimal result and it is better than the dynamic approach in terms of computational time and space complexity.

**Table 9:** Comparison of proposed model with dynamic programing.

No. of matrix	Sequence of dimensions	Optimal parenthesis	DP Multiplications	GCO Multiplications	Variation
10	5,10,21,78,12,15,20,18,6,22,25	(((((((((M1*M2)*M3)*M4)*M5)*M6)*M7)*M8)*M9)*M10)	22,070	22,070	0%
20	3,15,28,21,19,10,25,16,29,5,28,31,11,14,9,17,4,21,19,3,34	(((((((((((((((((M1*M2)*M3)*M4)*M5)*M6)*M7)*M8)*M9)*M10)*M11)*M12)*M13)*M14)*M15)*M16)*(M17*(M18*M19)))*M20)	15,909	15,909	0%
30	8,31,10,14,11,15,28,12,2,20,25,16,19,9,40,21,8,19,28,34,37,40,28,30,29,45,13,20,33,44,58	((M1*(M2*(M3*(M4*(M5*(M6*(M7*M8)))))))*(((((((((((((((((((((M9*M10)*M11)*M12)*M13)*M14)*M15)*M16)*M17)*M18)*M19)*M20)*M21)*M22)*M23)*M24)*M25)*M26)*M27)*M28)*M29)*M30))	37,996	37,996	0%
40	8,31,10,14,11,15,28,12,2,20,25,16,19,9,40,21,8,19,28,34,37,3,15,28,21,19,10,25,16,29,5,28,31,11,14,9,17,42,21,19,53	((M1*(M2*(M3*(M4*(M5*(M6*(M7*M8)))))))*(((((((((((((((((((((((((((((((M9*M10)*M11)*M12)*M13)*M14)*M15)*M16)*M17)*M18)*M19)*M20)*M21)*M22)*M23)*M24)*M25)*M26)*M27)*M28)*M29)*M30)*M31)*M32)*M33)*M34)*M35)*M36)*M37)*M38)*M39)*M40))	31,260	31,260	0%
50	5,6,2,13,24,5,16,18,13,4,11,31,15,13,14,10,15,13,18,19,14,15,13,23,44,12,9,26,6,14,32,19,22,32,2,21,11,12,25,19,20,33,22,32,77,21,34,44,26,43,32	(((M1*M2)*(((((((M3*M4)*M5)*M6)*M7)*M8)*M9)*(M10*(M11*(M12*(M13*(M14*(M15*(M16*(M17*(M18*(M19*(M20*(M21*(M22*(M23*(M24*(M25*(M26*(M27*(M28*(M29*(M30*(M31*(M32*(M33*M34))))))))))))))))))))))))))*(((((((((((((((M35*M36)*M37)*M38)*M39)*M40)*M41)*M42)*M43)*M44)*M45)*M46)*M47)*M48)*M49)*M50))	44,778	44,778	0%
58	6,2,13,24,5,16,18,13,4,11,31,15,13,14,10,15,13,18,19,14,15,13,23,44,12,9,26,6,4,2,22,32,32,2,21,11,12,25,19,20,33,22,32,21,34,44,26,43,32,33,22,32,21,34,44,26,43,32,78	((M1*((((((((M2*M3)*M4)*M5)*M6)*M7)*M8)*(M9*(M10*(M11*(M12*(M13*(M14*(M15*(M16*(M17*(M18*(M19*(M20*(M21*(M22*(M23*(M24*(M25*(M26*(M27*(M28*M29)))))))))))))))))))))*(M30*(M31*(M32*M33)))))*((((((((((((((((((((((((M34*M35)*M36)*M37)*M38)*M39)*M40)*M41)*M42)*M43)*M44)*M45)*M46)*M47)*M48)*M49)*M50)*M51)*M52)*M53)*M54)*M55)*M56)*M57)*M58))	60,600	60,600	0%

To check and investigate the performance of proposed model, we also compare it with arithmetic approach. The comparison between proposed model and Arithmetic Approach demonstrated in Table 10, the results of arithmetic multiplication get form the published article (Hafeez et al., 2007), which describes that the results are optimal for the used data set, we get the same data set and apply the proposed model. The results in the Table 10 show that both the arithmetic multiplication and proposed model generated the same results, this proves that the proposed model provides the optimal results. But the proposed model perform better than the arithmetic multiplication approach in terms of computational time and space complexity as shown in the Table 7.

**Table 10:** Comparison of proposed model with arithmetic multiplications.

No. of matrix	Sequence of dimensions	Optimal parenthesis	Arithmetic multiplications	GCO multiplications	Variation (%)
3	9,95,21,78	((M1*M2)*M3)	32,697	32,697	0
6	30,10,71,58,9,25,22	(M1*((M2*(M3*M4))*(M5*M6)))	56,982	56,982	0
9	94,67,56,17,80,68,10,78,7,5	(M1*(M2*(M3*(M4*(M5*(M6*((M7*M8)*M9)))))))	98,220	98,220	0
12	42,54,49,22,62,46,93,97,82,59,24,86,56	(((M1*(M2*M3))*((((((M4*M5)*M6)*M7)*M8)*M9)*M10))*(M11*M12))	970,214	970,214	0
15	27,98,89,40,36,82,6,11,3,23,15,91,87,35,3,43	((M1*(M2*(M3*(M4*(M5*(M6*((M7*M8)*((M9*M10)*(M11*(M12*(M13*M14)))))))))))*M15)	101,322	101,322	0
18	94,30,63,79,52,10,6,13,93,97,3,8,67,40,38,6,89,61,71	((M1*(M2*(M3*(M4*(M5*(M6*(M7*(M8*(M9*M10)))))))))*(((((((M11*M12)*M13)*M14)*M15)*M16)*M17)*M18))	139,845	139,845	0
21	57,92,76,77,28,13,47,27,3,67,89,14,93,16,24,34,14,83,8 9,92,33,19	((M1*(M2*(M3*(M4*(M5*(M6*(M7*M8)))))))*((((((((((((M9*M10)*M11)*M12)*M13)*M14)*M15)*M16)*M17)*M18)*M19)*M20)*M21))	166,938	166,938	0
24	79,68,62,22,98,35,62,99,21,39,91,79,81,31,11,4,87,90,90,72,57,92,36,72,59	((M1*(M2*(M3*(M4*(M5*(M6*(M7*(M8*(M9*(M10*(M11*(M12*(M13*(M14*M15))))))))))))))*((((((((M16*M17)*M18)*M19)*M20)*M21)*M22)*M23)*M24))	377,216	377,216	0

The Table 11 shows the console output of the resultant matrix execution time, where dimension size of the matrices varies from 1 to 100.

**Table 11:** Execution time of proposed model.

No. of matrix	Optimal structure (Parenthesis)	Optimal multiplication	Time of execution (S)
50	((M1*(M2*(M3*(M4*(M5*(M6*(M7*(M8*(M9*(M10*(M11*(M12*(M13*(M14*(M15*(M16*(M17*(M18*(M19*(M20*(M21*(M22*(M23*(M24*(M25*(M26*(M27*(M28*(M29*(M30*(M31*(M32*(M33*(M34*(M35*(M36*M37))))))))))))))))))))))))))))))))))))*((((((((((((M38*M39)*M40)*M41)*M42)*M43)*M44)*M45)*M46)*M47)*M48)*M49)*M50))	458,949	3.15
40	((M1*(M2*(M3*(M4*(M5*(M6*(M7*(M8*(M9*(M10*(M11*(M12*(M13*(M14*(M15*(M16*(M17*(M18*(M19*(M20*(M21*(M22*(M23*(M24*(M25*(M26*(M27*(M28*(M29*(M30*(M31*(M32*(M33*(M34*(M35*(M36*M37))))))))))))))))))))))))))))))))))))*((M38*M39)*M40))	428,912	2.55
30	((M1*(M2*(M3*(M4*(M5*(M6*(M7*(M8*(M9*(M10*(M11*(M12*(M13*(M14*(M15*(M16*(M17*(M18*M19))))))))))))))))))*((((((((((M20*M21)*M22)*M23)*M24)*M25)*M26)*M27)*M28)*M29)*M30))	345,560	2.06
20	((M1*(M2*(M3*(M4*(M5*(M6*(M7*(M8*(M9*(M10*(M11*(M12*(M13*(M14*(M15*(M16*(M17*(M18*M19))))))))))))))))))*M20)	236,664	1.75
10	(((((((((M1*M2)*M3)*M4)*M5)*M6)*M7)*M8)*M9)*M10)	256,527	1.02

## Concluding Remarks

This research concludes that the GCO can enhance the power of simple dynamic programing problems by reducing its space and time complexity at a great extent. Moreover, the use of GCO algorithm also reduces the arithmetic multiplication operations for CMMP. The experimental results shows that our enhanced CMM version based on GCO provide good performance and reduce the time for matrix multiplication from 45% to 96% when compared with sequential multiplication. Moreover, we evaluate our results with the best known dynamic programing arithmetic multiplication approach which clearly demonstrate that proposed model outperforms in terms of computational time and space complexity. We have also identified that when we minimize the required operation for CMM operation, the number of resources increases and it requires higher data throughput bandwidth. Fine grain nature of matrix multiplication problem through dynamic programing; the 50 matrix chain product problem was solved on one processor. One of the major drawback of DP approach is that it requires number of processors equal to the number of matrices in parallel computing is a difficult task to fulfill in most of the cases. The proposed model compared with other existing approach of multiplication and shows that our proposed approach has better optimal solution.

## Supplemental Information

10.7717/peerj-cs.395/supp-1 Supplemental Information 1 Dataset Summary.

10.7717/peerj-cs.395/supp-2 Supplemental Information 2 Source Code.

## Additional Information and Declarations

### Data Availability

The following information was supplied regarding data availability: Source codes are available in the Supplemental Files.
