# Supplementary material for: Optimal sequence for chain matrix multiplication using evolutionary algorithm
Source: PeerJ Comput Sci. 2021 Feb 26;7:e395. doi: 10.7717/peerj-cs.395 (PMC7959611; doi:10.7717/peerj-cs.395)
Supplement: Supplemental Information 1 [file peerj-cs-07-395-s001.docx]

| **No of matrix**  **Summary of Dataset 1** | **Sequence of Dimensions**  **(Dimensions of Matrices)** |
| --- | --- |
| 9 | 94,67,56,17,80,6 8,10,78,7,5 |
| 12 | 42,54,49,22,62,46,93,97,82,59,24,86,56 |
| 15 | 27,98,89,40,36,82,6,11,3,23,15,91 ,87,35,3,43 |
| 18 | 94,30,63,79,52,10,6,13,93,97,3,8,67,40,38,6,89,61, 71 |
| 21 | 57,92,76,77,28,13,47,27,3,67,89,4,93,16,24,4,14,83,89,92,33,19 |
| 24 | 79,68,62,22,98,35,62,99,21,39,91,79,81,31,11,4,87,90,90,72,57,92,3 6,72,59 |
| 30 | 50,44,56,33,44,5,9,10,12,22,32,26,41,28,19,29,41,23,18,25,22,34,33,13,33,11,43,21,2 4,56,71 |
| 50 | 56,34,33,46,39,50,65,32,10,15,30,24,25,13,7,11,19,30,15,3,20,31,50,9,10,16,44,22,10,16,44,22,10,19,30,40,45,23,22,14, 30,11,22,24,32,15,19,29,34,5,9,23 ,29,34,9 |

| **No of matrix** | **Sequence of dimensions**  **(Dimensions of Matrices)** |
| --- | --- |
| 10 | 5, 10, 21, 78, 12, 15, 20, 18, 6, 22, 25 |
| 20 | 3, 15, 28, 21, 19, 10, 25, 16, 29, 5, 28, 31,11, 14, 9, 17, 4, 21, 19, 3, 34 |
| 30 | 8, 31, 10, 14 ,11,15, 28,12,2,20,25,16,19,9,40,21,8,19,28,34,37,40,28,30,29,45,13,20,33,44,58 |
| 40 | 8, 31, 10, 14, 11,15,28,12,2,2 0,25,16,19,9,40 ,21,8, 19, 28,34, 37, 3, 15, 28, 21, 19, 10, 25, 16, 29, 5, 28, 31, 11, 14, 9, 17, 42, 21, 19, 53 |
| 50 | 5,6,2,13,24,5,1 6,18,13,4,11,31 ,15,13,14,10,15 ,13,18,19,14,15 ,13,23,44,12,9, 26,6,14,32,19,2 2,32,2,21,11,12 ,25,19,20,33,22 ,32,77,21,34,44 ,26,43,32 |
| 58 | 6,2,13,24,5,16, 18,13,4,11,31,1 5,13,14,10,15,1 3,18,19,14,15,1 3,23,44,12,9,26 ,6,4,2,22,32,32, 2,21,11,12,25,1 9,20,33,22,32,2 1,34,44,26,43,3 2,33, 22, 32, 21, 34, 44, 26, 43, 32, 78 |

**Summary of Dataset 2**

**Summary of Dataset 3**

| **No of matrix** | **Sequence of dimensions**  **(Dimensions of Matrices)** |
| --- | --- |
| 3 | 9,95,21,78 |
| 6 | 30,10,71,58,9,25,22 |
| 9 | 94,67,56,17,80,68,10,78,7,5 |
| 12 | 42,54,49,22,62,46,93,97,82, 59,24,86,56 |
| 15 | 27,98,89,40,36,82,6,11,3,23, 15,91,87,35,3,43 |
| 18 | 94,30,63,79,52,10,6,13,93,9 7,3,8,67,40,38,6,89,61,71 |
| 21 | 57,92,76,77,28,13,47,27,3,6 7,89,14,93,16,24,34,14,83,8 9,92,33,19 |
| 24 | 79,68,62,22,98,35,62,99,21, 39,91,79,81,31,11,4,87,90,9 0,72,57,92,36,72,59 |
